# 微波消融治疗肺癌的免疫机制进展

**DOI:** 10.3779/j.issn.1009-3419.2025.106.30

**Published:** 2025-11-20

**Authors:** YANG Zhenyu, CHEN Xiaowen, JIANG Junhong

**Affiliations:** 215000 苏州，苏州大学附属第四医院（苏州市独墅湖医院） 呼吸与危重症医学科; Department of Respiratory and Critical Care Medicine, The Fourth Affiliated Hospital of Soochow University Suzhou Dushu Lake Hospital, Suzhou 215000, China

**Keywords:** 微波消融, 肺肿瘤, 免疫激活, 肿瘤免疫微环境, 免疫治疗, Microwave ablation, Lung neoplasms, Immune activation, Tumor immune microenvironment, Immunotherapy

## Abstract

微波消融（microwave ablation, MWA）是一种微创的热消融治疗手段，目前已广泛应用于肺癌的局部治疗。MWA不仅能够局部有效地诱导肿瘤细胞坏死，还能通过调控肿瘤免疫微环境（tumor immune microenvironment, TIME）诱发潜在远端效应来激活抗肿瘤免疫效应；此外，MWA联合抗肿瘤免疫药物可能发挥协同效应，这为肺癌患者的治疗提供了新的方向。本文综述了MWA治疗肺癌过程中抗肿瘤免疫反应激活的机制，旨在为肺癌未来的治疗策略提供理论基础和实践指导。

## Abstract (en)

Microwave ablation (MWA) is a minimally invasive thermal ablation treatment method and has been widely applied in the local treatment of lung cancer. MWA not only can effectively induce local tumor cell necrosis, but also can activate anti-tumor immune responses by regulating the tumor immune microenvironment (TIME) and inducing potential distant effects. In addition, the combination of MWA and anti-tumor immune drugs may exert a synergistic effect, which provides a new direction for the treatment of lung cancer patients. This article reviews the mechanisms of activation of anti-tumor immune responses during MWA treatment of lung cancer, aiming to provide a theoretical basis and practical guidance for future treatment strategies of lung cancer.

肺癌是全球癌症相关死亡的主要原因，每年约有200万例新发病例和176万死亡病例；尽管外科手术、放化疗、靶向治疗和免疫治疗等现有手段使得肺癌患者显著获益，但晚期肺癌的生存率仍处于较低水平^[1]^。微波消融（microwave ablation, MWA）作为一种相对较新的局部热消融技术，随着国产化进程的推进已实现广泛应用；与射频消融（radiofrequency ablation, RFA）和冷冻消融相比，MWA具有操作时间更短、消融范围更大的优势，已成为肺癌局部治疗的重要选择^[2,3]^；除了直接破坏肿瘤外，MWA伴随的免疫反应也逐渐受到关注^[4]^。研究^[4,5]^表明，MWA能够通过诱导局部肿瘤细胞坏死而激活全身免疫反应，从而增强抗肿瘤免疫；此外，MWA与抗肿瘤免疫药物联合可发挥协同效应，显著改善晚期肺癌患者的预后^[6]^。基于此，本文综述了MWA治疗肺癌的免疫机制研究现状，旨在为肺癌综合治疗策略的优化提供研究方向。

## 1 MWA及其在肺癌治疗中的应用

### 1.1 MWA的原理和技术优势

目前常用的肿瘤介入治疗手段包括MWA、RFA、冷冻消融等，且均会引起不同程度的免疫激活；与RFA不同，MWA不依赖电流和组织传导，更适合于肺脏等阻抗较高、含水量较高的组织；相比RFA和冷冻消融，MWA可在更短的时间内实现更大的消融体积^[7,8]^。此外，MWA通过释放的高频电磁波可以使组织内的水分子快速振动，形成局部高温，从而导致肿瘤组织的蛋白质变性和细胞坏死；在此过程中，机体的免疫反应也被同步激活^[4,7,9]^。MWA为高龄、合并基础病无法耐受常规治疗的患者提供了新的选择。

### 1.2 MWA治疗肺癌的临床应用现状

目前，MWA已广泛应用于肺癌的局部治疗。一项前瞻性、单臂和单中心的临床研究^[10]^纳入97例70岁以上早期肺癌患者，经MWA治疗的1年总生存率和无进展生存率分别为99.0%和93.7%。但有证据^[11]^指出，人体衰老的过程中也会出现“免疫衰老”，表现为免疫抑制性增强，如CD8^+^ T细胞等抗肿瘤免疫细胞明显减少。这可能影响MWA的免疫激活效果。Wang等^[12]^开展的回顾性研究纳入231例早期非小细胞肺癌患者，均接受胸部计算机断层扫描（computed tomography, CT）引导下的MWA治疗，消融功率为（37.81±7.05）W，消融时间（6.20±1.59）min，所有的肿瘤均小于3 cm，其中66例肿瘤邻近支气管血管束，结果发现患者术后1、2和3年无进展生存率分别为77.4%、70.5%和63.8%；此外，单因素分析提示肿瘤大小大于2 cm、实性肿瘤、肿瘤邻近支气管血管束、以及消融边缘大于5 mm是局部进展的危险因素；多因素分析进一步表明，仅肿瘤邻近支气管血管束与患者无进展生存率显著降低有关。以上研究提示包括年龄、肿瘤大小、肿瘤位置和消融范围都可能与MWA的疗效或免疫效应存在潜在的联系。因而，单纯MWA治疗早期肺癌仍面临复发的风险，此外其治疗晚期肺癌疗效也有限^[13]^。近期的研究^[14,15]^表明，MWA与抗肿瘤免疫药物联合应用可降低肺癌复发的风险并提升治疗的效果。因此，深入阐明MWA诱导肺癌抗肿瘤免疫效应的机制，对进一步优化MWA治疗方案具有重要的意义。

## 2 MWA诱导的抗肿瘤免疫反应

### 2.1 MWA诱导的肿瘤细胞坏死和免疫反应激活

MWA作为一种局部热消融技术，其释放的微波能量被肿瘤组织吸收后可使组织温度迅速升高，直接导致肿瘤细胞坏死。这种热损伤除直接杀死肿瘤细胞外，还能诱导局部炎症微环境形成，促使中性粒细胞和单核细胞浸润至肿瘤组织，这些细胞也会释放各种细胞因子和化学物质，进一步激活肿瘤特异性T细胞^[4]^。更重要的是，MWA诱导的肿瘤细胞坏死具有免疫原性，即发生免疫原性细胞死亡。坏死的肿瘤细胞会释放热休克蛋白（heat shock proteins, HSPs）、三磷酸腺苷（adenosine triphosphate, ATP）、高迁移率族蛋白B1（high mobility group protein B1, HMGB1）等损伤相关分子模式（damage associated molecular patterns, DAMPs），而DAMPs是调控肿瘤免疫应答的关键分子^[16-18]^。研究^[19]^证实，细胞外的HSP70能够通过Toll样受体-2（Toll-like receptor-2, TLR-2）和TLR-4与抗原递呈细胞（antigen-presenting cells, APCs）结合，该结合可够触发髓样分化因子88（myeloid differentiation primary response 88, MyD88）依耐性信号传导的级联反应，激活核因子-κB（nuclear factor-κB, NF-κB）和有丝分裂原活化蛋白激酶（mitogen-activated protein kinase, MAPKs）；其中，NF-κB的激活会进一步促进促炎性细胞因子如肿瘤坏死因子-α（tumor necrosis factor-α, TNF-α）、白细胞介素（interleukin, IL）-1β的表达和分泌，从而增强APCs的活化。另有研究^[20]^表明ATP可以通过嘌呤能受体P2X7（purinergic receptor P2X7, P2RX7）和泛连接蛋白1（pannexin 1, PANX1）通路促进树突状细胞的活化和迁移。上述机制为MWA激活抗肿瘤免疫反应提供了重要的实验依据。

### 2.2 MWA对肿瘤免疫微环境（tumor immune microenvironment, TIME）的激活

尽管MWA是一种局部的治疗手段，但可引发全身的免疫反应。一项纳入45例肺癌患者的研究^[21]^显示，MWA治疗后24 h外周血CD8^+ ^T细胞的比例显著增高，同时IL-2、IL-1β、IL-6、IL-12p70、IL-22、TNF-α和干扰素-γ（interferon-γ, IFN-γ）等细胞因子水平明显升高，提示Th1型免疫反应被激活。另一项前瞻性研究^[22]^纳入22例肺癌患者，对比了MWA治疗后1天与1个月的免疫指标发现，术后1个月的CD8^+ ^T细胞比例显著升高，而具有免疫抑制性的调节性T细胞（regulatory T cells, Tregs）的比例显著降低；进一步分析显示，Tregs细胞比例降幅超过均值的患者，其中位无进展生存期明显长于降幅低于均值者。系统性炎症反应指数（systemic inflammatory response index, SIRI）基于外周血中的绝对淋巴细胞、单核细胞和中性粒细胞计数计算得出，是肺癌预后预测的有效工具^[23]^；Wang等^[24]^对接受MWA治疗的265例早期非小细胞肺癌患者的研究发现，SIRI预测总体生存率和无进展生存率的受试者工作特征曲线的曲线下面积分别为0.796和0.716，Cox比例风险模型显示，高SIRI水平与总体生存率和无进展生存率显著降低存在独立关联，进一步印证了MWA治疗与机体免疫和炎症反应的密切关系。

在实体肿瘤发展的早期，肿瘤引流淋巴结（tumor-draining lymph node, TdLN）中抗原递呈是建立免疫监视的关键过程，TdLN可以促进全身肿瘤特异性T细胞反应并与免疫治疗的反应密切关联^[25,26]^。动物实验^[27]^表明，MWA可以通过改变TdLN中的TIME而增强抗肿瘤免疫反应：在小鼠肺癌模型中，MWA局部治疗后TdLN中CD4^+ ^T细胞和自然杀伤细胞的比例显著增加，Tregs细胞的比例则显著降低。TIME主要由肿瘤细胞、免疫细胞和细胞因子等组成，其与机体的抗肿瘤免疫亦密切关联。TIME中的免疫细胞分为两类，即肿瘤免疫促进性细胞和肿瘤免疫抑制性细胞，肿瘤免疫促进性细胞主要有细胞毒性CD8^+ ^T细胞、效应CD4^+ ^T细胞、树突状细胞和M1极化的巨噬细胞等；肿瘤免疫抑制性细胞主要有Tregs细胞、骨髓来源的抑制性细胞（myeloid-derived suppressor cells, MDSCs）、M2极化巨噬细胞等。TIME中两种类型的免疫细胞的平衡直接决定了抗肿瘤免疫效应^[28,29]^。在一项乳腺癌的动物研究^[30]^中学者发现RFA联合免疫治疗可以增加肿瘤内M1极化的巨噬细胞、效应CD4^+ ^T细胞和细胞毒性CD8^+ ^T细胞的比例，并可以显著抑制原发肿瘤生长、未治疗的远处肿瘤生长以及肿瘤肺转移。尽管肿瘤存在异质性，但TIME在肿瘤进展中的作用具有共性^[29]^。Guo等^[31]^的研究证实，MWA可以增加肺癌组织中局部T细胞的丰度并改变单核细胞的相互作用，从而调控TIME。另有研究^[6]^指出，MWA联合程序性细胞死亡受体-1（programmed cell death protein-1, PD-1）抑制剂可通过刺激细胞因子如IFN-γ和TNF-α的释放，促进自然杀伤细胞、巨噬细胞等免疫细胞的活化，增加CD8^+^ T细胞的浸润，实现TIME重塑并提升肺癌治疗过程中的抗肿瘤免疫反应。小鼠肺癌模型^[32]^进一步发现，MWA治疗背部单侧肿瘤可以抑制对侧肿瘤的生长，且MWA联合PD-1抑制剂可以通过CXC趋化因子配体10（C-X-C motif chemokine ligand 10, CXCL10）通路，增加肿瘤内CD45^+^和IFN-γ^+^的CD8^+ ^T细胞的浸润，显著提高治疗效果。然而，MWA相关免疫反应激活的机制仍处于初步研究阶段，诸如MWA对TIME中M1极化巨噬细胞、MDSCs等的调控机制仍需进一步研究。

### 2.3 MWA诱导的免疫机制与临床疗效关联性

如前所述，MWA可以激活抗肿瘤免疫反应，近年来的多项研究进一步阐明了“MWA-免疫激活-临床疗效”的关联机制。Tregs细胞作为肿瘤免疫抑制性细胞，可通过干扰抗原递呈和T细胞活化、抑制效应T细胞功能或促进其耗竭、直接破坏效应T细胞等介导免疫抑制^[33]^。有学者^[22]^发现MWA治疗可以降低肺癌患者外周血Tregs细胞的水平，且Tregs降幅超过均值的患者无进展生存期明显更长，直接证实Tregs水平与MWA治疗疗效的关联性。肿瘤的复发与播散肿瘤细胞（disseminated tumor cells, DTCs）密切关联，而Th1型免疫反应的激活可以通过调控DTCs来抑制肿瘤的复发和转移^[34]^。已有研究^[21]^证实MWA可以激活肺癌患者的Th1型免疫反应，但MWA治疗是否能够通过Th1型免疫反应的激活以降低肿瘤的复发和转移仍需更多的临床和基础研究验证。

## 3 MWA诱导的远端效应

远端效应指局部MWA治疗后，未直接消融的远处肿瘤（包括区域淋巴结转移灶及远处器官转移灶）出现体积缩小或消退的现象，提示局部介入治疗激活了全身的抗肿瘤免疫反应^[35-37]^。临床病例报道已初步观察到MWA的远端效应。1例晚期肺鳞癌患者在接受肺部病灶的MWA治疗后，纵隔淋巴结的转移病灶也出现明显缩小^[38]^。另有1例原发子宫内膜癌合并双侧肺转移的女性患者，仅对右肺的1个病灶实施MWA治疗，右肺其他病灶也逐渐消退^[39]^。Cao等^[37]^开展的大样本研究纳入540例原发或转移性肺癌或肝癌患者，均接受MWA治疗，结果显示13例患者出现远端效应，其外周血中单核细胞/巨噬细胞和T细胞比例明显高于未出现远端效应的患者，但在该研究中远端效应发生率较低（约2.4%，13/540），且缺乏对照组数据，无法排除肿瘤自然消退的可能性。在小鼠肺癌模型中MWA联合嵌合受体免疫疗法（chimeric antigen receptor T-cell immunotherapy, CAR-T）可以通过增强CAR-T细胞的活化，在肿瘤中的浸润以及耗竭的减少促进抗肿瘤免疫来显著抑制原发和转移肿瘤^[40]^。尽管支撑MWA在肺癌中远端效应的临床及基础研究数据仍较有限，但也提示MWA具备激活全身免疫反应并抑制远处肿瘤的潜力，未来需开展设计严谨的对照研究，进一步验证MWA的远端效应并阐明其分子机制。

## 4 MWA与抗肿瘤免疫药物联合治疗的前景

尽管MWA能够局部杀死肺癌细胞，并在一定程度上可以激活抗肿瘤免疫反应，但其触发的免疫反应强度仍有限，疗效有待优化。一项纳入12项研究、985例患者的系统综述^[13]^显示，1336例肺恶性结节接受MWA治疗的复发率达到9%-37%。这凸显了单纯MWA治疗的局限性，MWA与免疫检查点抑制剂的免疫协同效应为肺恶性肿瘤的治疗提供了新的方向。临床研究已初步证实MWA与PD-1抑制剂联合的治疗价值。1例PD-1抑制剂耐药后的晚期鳞癌患者在接受肺部病灶MWA治疗后，术后1个月不仅消融的肺部病灶吸收，转移性的纵隔淋巴结也明显缩小，截止随访时间，重新启用PD-1抑制剂治疗的患者的无进展生存期约为6个月，提示患者从原有PD-1抑制剂治疗中重新获益^[38]^。在1项纳入21例晚期非小细胞肺癌患者的前瞻性临床研究^[14]^中，MWA治疗后的第5-7天联合卡瑞利珠单抗（200 mg/次/2周）治疗，终点为疾病进展或出现不可耐受的毒性反应，结果显示，客观缓解率为33.3%，2例患者达到完全缓解，5例患者达到部分缓解，中位无进展生存期为5.1个月；这21例接受联合治疗的患者，有8例出现了2级不良反应，3例出现了3级不良反应。Xu等^[6]^开展的研究纳入62例IIIB-IV期的非小细胞肺癌患者，其中30例接受了卡瑞丽珠单抗单药治疗，32例接受了卡瑞丽珠单抗联合MWA治疗；结果显示，联合治疗的客观缓解率（43.8% vs 16.7%）和无进展生存期（9.88 vs 5.32个月）明显优于PD-1抑制剂单药。但以上研究是基于小样本或单中心的研究，可能存在选择偏倚，需多中心更大样本量的随机对照试验和基础实验去进一步探究MWA联合抗肿瘤免疫药物的协同作用和机制。Xu等^[6]^后续的动物实验则揭示了MWA和抗肿瘤免疫药物的协同机制：MWA联合PD-1抑制剂可以提高TdLN和肿瘤中CD8^+ ^T细胞的功能和比例，还可以提高中央记忆T细胞的比例；而中央记忆T细胞是具有长期记忆性的，并能够归巢到淋巴结接受抗原再刺激的T细胞，具有强效的抗肿瘤能力。除PD-1抑制剂外，MWA与其他免疫调节剂的联合方案也取得初步进展。CpG顺序特异性寡核苷酸（CpG oligodeoxynucleotides, CpG ONDs）作为一种TLR-9激动剂，可激活TLR-9/MyD88/干扰素调节因子7（interferon regulatory factor 7, IRF7）和TLR-9/MyD88/NF-κB信号通路，从而诱导天然免疫细胞产生I型IFN和促炎性细胞因子，进而启动Th1型免疫反应和特异性T细胞反应^[41]^。小样本临床研究^[42]^也表明CpG ONDs可以改善PD-1抑制剂耐药。一项临床前动物研究^[15]^表明，MWA联合CpG ONDs和PD-1抑制剂可以显著增加肺癌组织和脾脏中的CD8^+ ^T细胞数量，减少Tregs细胞的数量；此外，MWA联合CpG ONDs和PD-1抑制剂还可以显著升高血浆中的TNF-α和IFN-γ的浓度，并降低转化生长因子-β（transforming growth factor-β, TGF-β）的浓度，从而抑制肺癌的生长。不同于传统的抗肿瘤免疫药物，FMS样酪氨酸激酶3配体（FMS-like tyrosine kinase 3 ligand, FLT3L）是一种细胞因子，其属于III型受体酪氨酸激酶家族，广泛表达于造血干细胞和祖细胞中，近年来也被发现具有免疫调节功能，能以依赖于Janus激酶（Janus kinase, JAK）/信号转导及转录激活因子1（signal transducer and activator of transcription 1, STAT1）信号通路诱导出CD44高表达的CD8^+ ^T细胞^[43-45]^。在小鼠肺癌模型中，MWA联合FLT3L可以通过促进TdLN中的肿瘤特异性记忆T细胞的增殖和分化以及抑制TIME中的T细胞耗竭来显著抑制肿瘤复发，并可以显著改善PD-1抑制剂的耐药^[46]^。MWA激活抗肿瘤免疫反应的示意图见图1^[4,6,7,9,16-20,25-29,31,32,41-46]^。目前，MWA联合免疫治疗的作用机制尚未完全阐明，如不同联合方案中免疫细胞亚群的精准调控机制、协同效应的关键调控节点等仍需深入探索。此外，联合方案的临床优化如免疫药物的给药方式（局部或全身）、MWA与免疫治疗的给药顺序和时间间隔以及不良反应管理等问题，也有待更多临床研究明确。

**图 1:**
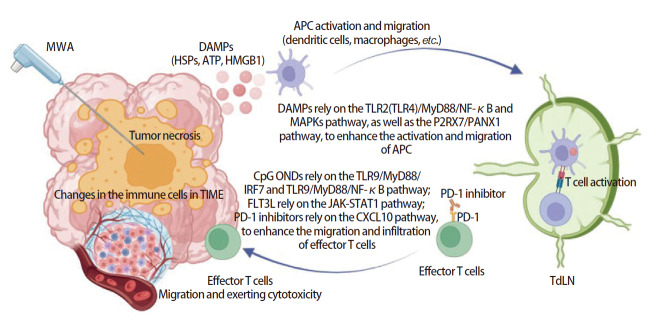
MWA激活抗肿瘤免疫反应的示意图[Created in BioRender. Yang, Z. (2025), https://BioRender.com/hekvab4]

## 5 机制导向的MWA联合治疗方案设计

在肺癌治疗过程中，MWA联合免疫治疗可以通过调控抗肿瘤免疫反应来发挥协同效应，也为开发新的联合治疗策略提供了参考。小鼠肺癌模型研究^[32]^证实，MWA联合PD-1抑制剂可以通过CXCL10通路调控肺癌组织中抗肿瘤免疫细胞的浸润，以显著增强疗效，该研究明确提示CXCL10通路是MWA与免疫治疗协同作用的关键点，为后续设计MWA联合CXCL10激动剂的基础研究和临床试验提供了直接的理论依据。FLT3L作为一种能够调节免疫功能的细胞因子，其已被证实可以通过JAK/STAT1信号通路诱导CD44高表达的CD8^+ ^T细胞^[43-45]^；而小鼠肺癌模型^[46]^显示，MWA联合FLT3L可以明显提高对肿瘤的疗效，提示靶向JAK/STAT1信号通路可能增强MWA的免疫激活效应，为未来开发MWA联合JAK/STAT1信号通路激动剂的治疗方案提供了重要研究方向。需注意的是，目前MWA联合免疫治疗的相关研究仍处于初步探索阶段，其协同作用的复杂机制及潜在联合策略尚未完全明确，仍需更多针对性研究深入挖掘。

## 6 未来与展望

近年来，随着呼吸介入技术的不断发展，MWA作为局部热消融技术已在肺癌治疗中实现广泛应用，但其疗效仍有进一步提升的空间。MWA通过诱导肿瘤细胞死亡，改变TIME中免疫细胞构成，诱发潜在远端效应来激活免疫效应，同时可能协同肿瘤免疫疗法实现更佳的临床预后，为肺癌患者的治疗提供了新的方向。然而，目前针对MWA激活抗肿瘤免疫反应、调控TIME等研究仍处于初步阶段，未来还需要更多的基础研究以及更大规模的前瞻性临床研究去探讨MWA相关抗肿瘤免疫反应的复杂机制和MWA联合免疫治疗的临床有效性、安全性等，以期为肺癌治疗的新手段提供理论和实践依据。

## References

[1] Thai AA, Solomon BJ, Sequist LV, et al. Lung cancer. Lancet, 2021, 398(10299): 535-554. doi: 10.1016/S0140-6736(21)00312-3 34273294

[2] Li H, Ye X. Ten-year status of microwave ablation therapy research for lung cancer. Shiyong Zhongliu Zazhi, 2024, 39(4): 291-298.

[3] Bartlett EC, Rahman S, Ridge CA. Percutaneous image-guided thermal ablation of lung cancer: what is the evidence?. Lung Cancer, 2023, 176: 14-23. doi: 10.1016/j.lungcan.2022.12.010 36571982

[4] Xu F, Wei Z, Ye X. Immunomodulatory effects of microwave ablation on malignant tumors. Am J Cancer Res, 2024, 14(6): 2714-2730. doi: 10.62347/QJID8425 39005685 PMC11236778

[5] Sang J, Ye X. Potential biomarkers for predicting immune response and outcomes in lung cancer patients undergoing thermal ablation. Front Immunol, 2023, 14: 1268331. doi: 10.3389/fimmu.2023.1268331 38022658 PMC10646301

[6] Xu F, Sang J, Wang N, et al. Microwave ablation combined with immune checkpoint inhibitor enhanced the antitumor immune activation and memory in rechallenged tumor mouse model. Cancer Immunol Immunother, 2025, 74(5): 161. doi: 10.1007/s00262-025-04003-5 40131498 PMC11937475

[7] Chu KF, Dupuy DE. Thermal ablation of tumours: biological mechanisms and advances in therapy. Nat Rev Cancer, 2014, 14(3): 199-208. doi: 10.1038/nrc3672 24561446

[8] Lassandro G, Picchi SG, Corvino A, et al. Ablation of pulmonary neoplasms: review of literature and future perspectives. Pol J Radiol, 2023, 88: e216-e224. doi: 10.5114/pjr.2023.127062 37234463 PMC10207320

[9] Hong ZQ, Jin DC, Bai XD, et al. Research advances in thermal ablation for lung cancer. Zhongguo Xiongxinxueguan Waike Linchuang Zazhi, 2024, 31(1): 166-172.

[10] Peng JZ, Wang CE, Bie ZX, et al. Microwave ablation for inoperable stage I non-small cell lung cancer in patients aged ≥70 years: a prospective, single-center study. J Vasc Interv Radiol, 2023, 34(10): 1771-1776. doi: 10.1016/j.jvir.2023.06.014 37331589

[11] Dolan M, Libby KA, Ringel AE, et al. Ageing, immune fitness and cancer. Nat Rev Cancer, 2025, 25(11): 848-872. doi: 10.1038/s41568-025-00858-z 40813902

[12] Wang J, Li B, Zhang L, et al. Safety and local efficacy of computed tomography-guided microwave ablation for treating early-stage non-small cell lung cancer adjacent to bronchovascular bundles. Eur Radiol, 2024, 34(1): 236-246. doi: 10.1007/s00330-023-09997-z 37505251

[13] Nelson DB, Tam AL, Mitchell KG, et al. Local recurrence after microwave ablation of lung malignancies: a systematic review. Ann Thorac Surg, 2019, 107(6): 1876-1883. doi: 10.1016/j.athoracsur.2018.10.049 30508527

[14] Wei Z, Yang X, Ye X, et al. Camrelizumab combined with microwave ablation improves the objective response rate in advanced non-small cell lung cancer. J Cancer Res Ther, 2019, 15(7): 1629-1634. doi: 10.4103/jcrt.JCRT_990_19 31939448

[15] Yu Y, Niu F, Sun B, et al. Preclinical study of TLR stimulation combined PD-1 antibody enhance the therapeutic effect of microwave ablation on NSCLC. Clin Transl Oncol, 2025, 27(7): 2982-2992. doi: 10.1007/s12094-024-03820-x 39702688

[16] Senders ZJ, Martin RCG 2nd. Intratumoral immunotherapy and tumor ablation: a local approach with broad potential. Cancers (Basel), 2022, 14(7): 1754. doi: 10.3390/cancers14071754 35406525 PMC8996835

[17] Wei Z, Yu X, Huang M, et al. Nanoplatforms potentiated ablation-immune synergistic therapy through improving local control and suppressing recurrent metastasis. Pharmaceutics, 2023, 15(5): 1456. doi: 10.3390/pharmaceutics15051456 37242696 PMC10224284

[18] Lin H, Xiong W, Fu L, et al. Damage-associated molecular patterns (DAMPs) in diseases: implications for therapy. Mol Biomed, 2025, 6(1): 60. doi: 10.1186/s43556-025-00305-3 40877572 PMC12394712

[19] Zhang B, Qi R. The dual-function of HSP70 in immune response and tumor immunity: from molecular regulation to therapeutic innovations. Front Immunol, 2025, 16: 1587414. doi: 10.3389/fimmu.2025.1587414 40297581 PMC12034705

[20] Kepp O, Bezu L, Yamazaki T, et al. ATP and cancer immunosurveillance. EMBO J, 2021, 40(13): e108130. doi: 10.15252/embj.2021108130 34121201 PMC8246257

[21] Ma F, Lin Y, Ni Z, et al. Microwave ablation enhances the systemic immune response in patients with lung cancer. Oncol Lett, 2024, 27(3): 106. doi: 10.3892/ol.2024.14239 38298427 PMC10829076

[22] Zhang L, Zhang M, Wang J, et al. Immunogenic change after percutaneous microwave ablation in pulmonary malignancies: variation in immune cell subsets and cytokines in peripheral blood. Front Immunol, 2022, 13: 1069192. doi: 10.3389/fimmu.2022.1069192 36569954 PMC9780363

[23] Liu J, Li S, Zhang S, et al. Systemic immune-inflammation index, neutrophil-to-lymphocyte ratio, platelet-to-lymphocyte ratio can predict clinical outcomes in patients with metastatic non-small cell lung cancer treated with nivolumab. J Clin Lab Anal, 2019, 33(8): e22964. doi: 10.1002/jcla.22964 31282096 PMC6805305

[24] Wang J, Cui SP, Zhao Q, et al. Preoperative systemic immune-inflammation index-basednomogram for lung carcinoma following microwave ablation-a real world single center study. Front Oncol, 2024, 14: 1305262. doi: 10.3389/fonc.2024.1305262 38571504 PMC10987766

[25] Delclaux I, Ventre KS, Jones D, et al. The tumor-draining lymph node as a reservoir for systemic immune surveillance. Trends Cancer, 2024, 10(1): 28-37. doi: 10.1016/j.trecan.2023.09.006 37863720 PMC10843049

[26] Saddawi-Konefka R, Schokrpur S, Gutkind JS. Let it be: preserving tumor-draining lymph nodes in the era of immuno-oncology. Cancer Cell, 2024, 42(6): 930-933. doi: 10.1016/j.ccell.2024.05.015 38861928

[27] Sang J, Liu P, Wang M, et al. Dynamic changes in the immune microenvironment in tumor-draining lymph nodes of a lewis lung cancer mouse model after microwave ablation. J Inflamm Res, 2024, 17: 4175-4186. doi: 10.2147/JIR.S462650 38979433 PMC11228081

[28] Lv B, Wang Y, Ma D, et al. Immunotherapy: reshape the tumor immune microenvironment. Front Immunol, 2022, 13: 844142. doi: 10.3389/fimmu.2022.844142 35874717 PMC9299092

[29] Aliazis K, Christofides A, Shah R, et al. The tumor microenvironment’s role in the response to immune checkpoint blockade. Nat Cancer, 2025, 6(6): 924-937. doi: 10.1038/s43018-025-00986-3 40514448 PMC12317369

[30] Xu A, Zhang L, Yuan J, et al. TLR9 agonist enhances radiofrequency ablation-induced CTL responses, leading to the potent inhibition of primary tumor growth and lung metastasis. Cell Mol Immunol, 2019, 16(10): 820-832. doi: 10.1038/s41423-018-0184-y 30467420 PMC6804546

[31] Guo RQ, Li YM, Bie ZX, et al. Microwave ablation of non-small cell lung cancer enhances local T-cell abundance and alters monocyte interactions. BMC Cancer, 2025, 25(1): 605. doi: 10.1186/s12885-025-14002-5 40181307 PMC11966799

[32] Xiao W, Huang H, Zheng P, et al. The CXCL10/CXCR3 pathway contributes to the synergy of thermal ablation and PD-1 blockade therapy against tumors. Cancers (Basel), 2023, 15(5): 1427. doi: 10.3390/cancers15051427 36900218 PMC10000434

[33] Imianowski CJ, Chen Q, Workman CJ, et al. Regulatory T cells in the tumour microenvironment. Nat Rev Cancer, 2025, 25(9): 703-722. doi: 10.1038/s41568-025-00832-9 40494998

[34] Ramamoorthi G, Lee MC, Farrell CM, et al. Antitumor CD4^+^ T helper 1 cells target and control the outgrowth of disseminated cancer cells. Cancer Immunol Res, 2025, 13(5): 729-748. doi: 10.1158/2326-6066.CIR-24-0630 40249209 PMC12046335

[35] Faraoni EY, O'Brien BJ, Strickland LN, et al. Radiofrequency ablation remodels the tumor microenvironment and promotes neutrophil-mediated abscopal immunomodulation in pancreatic cancer. Cancer Immunol Res, 2023, 11(1): 4-12. doi: 10.1158/2326-6066.CIR-22-0379 36367967 PMC9808367

[36] Wu S, Jiao J, Wang N, et al. Tregs ST2 deficiency enhances the abscopal anti-tumor response induced by microwave ablation. Int Immunopharmacol, 2024, 143(Pt 2): 113330. doi: 10.1016/j.intimp.2024.113330 39423663

[37] Cao B, Liu M, Xiao Z, et al. CV1-secreting sCAR-T cells potentiate the abscopal effect of microwave ablation in heterogeneous tumors. Cell Rep Med, 2025, 6(2): 101965. doi: 10.1016/j.xcrm.2025.101965 39970874 PMC11866491

[38] Shao C, Yang M, Pan Y, et al. Case report: abscopal effect of microwave ablation in apatient with advanced squamous NSCLC and resistance to immunotherapy. Front Immunol, 2021, 12: 696749. doi: 10.3389/fimmu.2021.696749 34413851 PMC8368438

[39] Xu H, Sun W, Kong Y, et al. Immune abscopal effect of microwave ablation for lung metastases of endometrial carcinoma. J Cancer Res Ther, 2020, 16(7): 1718-1721. doi: 10.4103/jcrt.JCRT_1399_20 33565523

[40] Cao B, Liu M, Wang L, et al. Remodelling of tumour microenvironment by microwave ablation potentiates immunotherapy of AXL-specific CAR T cells against non-small cell lung cancer. Nat Commun, 2022, 13(1): 6203. doi: 10.1038/s41467-022-33968-5 36261437 PMC9581911

[41] Krieg AM. Therapeutic potential of Toll-like receptor 9 activation. Nat Rev Drug Discov, 2006, 5(6): 471-484. doi: 10.1038/nrd2059 16763660

[42] Otsuka T, Nishida S, Shibahara T, et al. CpG ODN (K3)-toll-like receptor 9 agonist-induces Th1-type immune response and enhances cytotoxic activity in advanced lung cancer patients: a phase I study. BMC Cancer, 2022, 22(1): 744. doi: 10.1186/s12885-022-09818-4 PMC9264631 35799134

[43] Tu HF, Kung YJ, Lim L, et al. FLT3L-induced virtual memory CD8 T cells engage the immune system against tumor. J Biomed Sci, 2024, 31(1): 19. doi: 10.1186/s12929-024-01006-9 38287325 PMC10826030

[44] Chun D, Park J, Lee S, et al. Flt3L enhances clonal diversification and selective expansion of intratumoral CD8^+^ T cells while differentiating into effector-like cells. Cell Rep, 2024, 43(12): 115023. doi: 10.1016/j.celrep.2024.115023 39616612

[45] Momenilandi M, Lévy R, Sobrino S, et al. FLT3L governs the development of partially overlapping hematopoietic lineages in humans and mice. Cell, 2024, 187(11): 2817-2837.e31. doi: 10.1016/j.cell.2024.04.009 38701783 PMC11149630

[46] Wang M, Sang J, Xu F, et al. Microwave ablation combined with FLT3L provokes tumor-specific memory CD8^+^ T cells-mediated antitumor immunity in response to PD-1 blockade. Adv Sci (Weinh), 2025, 12(4): e2413181. doi: 10.1002/advs.202413181 39629989 PMC11775548

